# Proteolytic and Structural Changes in Rye and Triticale Roots under Aluminum Stress

**DOI:** 10.3390/cells10113046

**Published:** 2021-11-05

**Authors:** Joanna Szewińska, Elżbieta Różańska, Ewa Papierowska, Mateusz Labudda

**Affiliations:** 1 Department of Biochemistry and Microbiology, Institute of Biology, Warsaw University of Life Sciences-SGGW, Nowoursynowska 159, 02-776 Warsaw, Poland; mateusz_labudda@sggw.edu.pl; 2 Department of Botany, Institute of Biology, Warsaw University of Life Sciences-SGGW, Nowoursynowska 159, 02-776 Warsaw, Poland; elzbieta_rozanska@sggw.edu.pl; 3 Water Centre, Warsaw University of Life Sciences-SGGW, Ciszewskiego 6, 02-776 Warsaw, Poland; ewa_papierowska@sggw.edu.pl

**Keywords:** abiotic stress, aluminum, heavy metal, phytocystatin, plant anatomy, protease inhibitor, protease, root border cell, *Secale cereale*, *Triticosecale*

## Abstract

Proteolysis and structural adjustments are significant for defense against heavy metals. The purpose of this study was to evaluate whether the Al^3+^ stress alters protease activity and the anatomy of cereale roots. Azocaseinolytic and gelatinolytic measurements, transcript-level analysis of phytocystatins, and observations under microscopes were performed on the roots of Al^3+^-tolerant rye and tolerant and sensitive triticales exposed to Al^3+^. In rye and triticales, the azocaseinolytic activity was higher in treated roots. The gelatinolytic activity in the roots of rye was enhanced between 12 and 24 h in treated roots, and decreased at 48 h. The gelatinolytic activity in treated roots of tolerant triticale was the highest at 24 h and the lowest at 12 h, whereas in treated roots of sensitive triticale it was lowest at 12 h but was enhanced at 24 and 48 h. These changes were accompanied by increased transcript levels of phytocystatins in rye and triticale-treated roots. Light microscope analysis of rye roots revealed disintegration of rhizodermis in treated roots at 48 h and indicated the involvement of root border cells in rye defense against Al^3+^. The ultrastructural analysis showed vacuoles containing electron-dense precipitates. We postulate that proteolytic-antiproteolytic balance and structural acclimation reinforce the fine-tuning to Al^3+^.

## 1. Introduction

Proteolysis, engaging a broad spectrum of hydrolytic enzymes called proteases (peptidases), is one of the most important biochemical processes required for protein metabolism of all living organisms [1]. In plants, these enzymes participate in many key physiological processes under both normal and stressful environmental conditions [2]. For example, during seed germination, proteases carry out degradation of storage proteins to provide the growing plants with nitrogen in the form of free amino acids and inorganic nitrogen compounds [3]. Proteases are necessary for the degradation of misfolded, damaged, and harmful proteins in the plant cells [4]. They are an essential component of the interaction between plants and phytopathogenic organisms [5,6]. Moreover, it has been shown that they fulfill important metabolic roles in plants under abiotic stresses such as heavy metals (HMs) [7,8], water deficit [9], waterlogging [10], salinity [11], or heat [12].

As uncontrolled proteolysis could be a serious threat to cells, plants, similar to other organisms, are equipped with a battery of direct and endogenous proteinaceous regulators of peptidase activity, namely peptidase inhibitors (PIs) [13]. PIs are classified according to their inhibition mechanism into competitive, non-competitive, uncompetitive, and suicide inhibitors or the kind of catalytic classes of endopeptidases that is inhibited (cysteine, serine, aspartic, and metallo-endopeptidases) [14]. Among these, plant PIs directed against cysteine endopeptidases, also known as phytocystatins (PhyCys), have been studied extensively, particularly with respect to their regulatory and defense functions in plants that grapple with abiotic [15] and biotic [16] stresses. Our previous articles have shown that PhyCys are essential for the balancing of protease activity during development and germination of cereal seeds [3] and for the response of triticale to drought stress [17]. This has inspired us to further explore the mechanisms underlying cereal responses to stress in the context of proteolysis regulation, and we present our latest discoveries related to it in this article.

Soil acidification is a natural phenomenon, but an intensive agriculture, and affected environmental and especially edaphic conditions linked with more and more noticeable worldwide climate alterations can increase it overwhelmingly [18]. Aluminum (Al) phytotoxicity is one of the major agronomic problems in acid soils. In these soils, Al comes in the form of [Al(H_2_O)_6_]^3+^ but also may be in the form of Al(OH)^2+^, Al(OH)_2_^+^, Al(OH)_3_^−^ and Al(OH)_4_^−^. At pH < 5.0, aluminum ions (Al^3+^) are released from inorganic and water-insoluble complex compounds into the soil solution and they can be rhizotoxic [19]. The first symptoms of Al toxicity on plants are a rapid inhibition of root growth and elongation. It has been shown that Al accumulates mainly in the apical parts of the roots (in the meristematic and the elongation zones), thus disturbing the differentiation of the meristematic tissue [20]. Al-damaged root system limits the uptake of water and nutrients, which leads to a wide range of biochemical, physiological, and structural (local and systemic) changes in plants and finally to poor yielding [21]. Among plant physiological processes that are affected under aluminum stress are the photosynthetic rate, stomatal conductance, transpiration, generation of reactive oxygen species, free calcium ions pool, plasmodesmal callose deposition, and mitochondrial respiration [21].

A toxic environment generated by Al^3+^ has forced plants to evolve survival strategies to reduce their toxicity. Two main mechanisms should be mentioned here: Al exclusion from the root zone and Al tolerance via accumulation in the symplast [22,23,24]. Interestingly, some species (e.g., buckwheat and tea plant) are very resistant to Al and can accumulate high concentrations of Al in the leaves, greater than 1% of their dry weight [23]. However, cereals such as rye (*Secale cereale* L.), maize (*Zea mays* L.), barley (*Hordeum vulgare* L.), or wheat (*Triticum aestivum* L.) rather use the Al exclusion mechanism by exuding organic acids (e.g., citrate, malate, and oxalate) from roots [25].

Rye is a species with high tolerance to diseases caused by pathogens and pests [26], and it is also recognized as one of the most Al-tolerant cereals [27,28], but studies on rye’s Al tolerance are still extremely restricted [29]. Among others, the above-mentioned genetic advantages of rye have been noticed and used to breed a new type of hybrid cereal crop—triticale (× *Triticosecale* Wittm. ex A. Camus). The hexaploid triticale, which is commonly grown today, contains a complex genome consisting of two wheat (AABB) genomes and one rye (RR) genome, and its tolerance to Al^3+^ toxicity is intermediate between wheat and rye. Moreover, it was shown that the cooperation of Al^3+^ tolerance genes from rye and wheat regulates triticale response against Al^3+^ toxicity [30,31].

There is fragmentary evidence in the literature that proteolytic changes are involved in the defense response of cereals to Al^3+^ toxicity [32,33,34]. Therefore, we hypothesized that the altered proteolytic response could play a role of immense importance in the cereal physiological mechanisms to resist Al^3+^ toxicity and damage. Here, we analyzed the tolerant rye cultivar “Dańkowskie Złote” and recognized it as a reference cereale species with high tolerance to Al^3+^. In addition, two cultivars of triticale which differ in their ability to tolerate Al^3+^, “KWS Trisol” (tolerant genotype) and “Subito” (sensitive genotype) have been evaluated. Based on published results by Niedziela et al. [25], we applied a such concentration of Al in medium (0.59 mM) that makes it possible to distinguish between tolerant and sensitive genotypes using a standard physiological test. Thus, the aim of this study was to examine the structural and metabolic responses of rye plants to Al^3+^ with emphasis on the proteolysis and compare it with the responses of two triticale cultivars, differing in their tolerance to Al^3+^. Moreover, it seemed particularly important to clarify whether high Al^3+^ tolerance of rye is correlated with activity of proteases balanced by PhyCys. To find that out, we used molecular and biochemical–physiological approaches in combination with microscopic methods.

## 2. Materials and Methods

### 2.1. Plant Material

The materials were roots of the Al^3+^ tolerant rye cultivar “Dańkowskie Złote” (DANKO Hodowla Roślin Sp. z o.o. in Choryń, Poland) and two triticale cultivars: “KWS Trisol” (Al^3+^ tolerant genotype) (KWS Lochow Poland Sp. z o.o. in Kondratowice) and “Subito” (Al^3+^ sensitive genotype) (DANKO Hodowla Roślin Sp. z o.o. in Choryń, Poland).

### 2.2. Plant’s Cultivation and Al^3+^ Tolerance Test

Cereals seeds were initially washed in tap water for 2 h. Then, they were surface decontaminated in 5% sodium hypochlorite with 0.2% Tween 20 for 10 min. Next, seeds were rinsed under tap water for 1 h. The decontaminated seeds (embryos upwards) were put into moist filter paper in Petri dishes (9 cm diameter) and kept at 4 °C for 24 h. Germinated seeds (separately for each species) were placed onto an aseptic medical gauze put on plastic test tube racks and placed in a tray filled with the standard Hoagland’s No. 2 medium (Sigma–Aldrich, Saint Louis, MO, USA). Plants were cultivated hydroponically with continuous aeration of the medium using an aquarium pump at controlled conditions: temperature (23 °C day/16 °C night), photoperiod (16 h day/8 h night), photosynthetic photon flux density (100 ± 25 μmol m^−2^ s^−1^) and 50% humidity in a growth chamber MLR-350 (Sanyo, Tokyo, Japan). After three days, half of the plants prepared for the experiments placed onto the same medium containing Al^3+^ (0.59 mM) in the form of AlCl_3_ and then rye and triticale roots were collected after 12, 24, and 48 h. The appropriate controls of Al^3+^ untreated cereal roots were sampled as well.

The physiological test for the evaluation of Al^3+^ tolerance described by Anioł [35] was applied. Up to the stage of introducing Al^3+^ into the medium, the growing conditions of the plants were the same as described above with such a difference that plants were placed onto the Hoagland’s medium containing Al^3+^ (0.59 mM) in the form of AlCl_3_ for 24 h. Then, rye and triticale roots were washed in deionized water and plants were placed again in the Hoagland’s medium for 48 h but without Al^3+^. To determine Al^3+^ tolerance of individual cereals, the root regrowth after their staining with 0.1% Eriochrome Cyanine R dye for Al (Sigma–Aldrich) for 10 min was measured. An exposition of roots to Al^3+^ resulted in irreversible damage of apical meristem of roots. The dye bound to these areas and dark purple color in damaged roots was visible. Tolerant cereal plants preserved their ability to continue root growth after removal of Al^3+^ from the medium. The root regrowth was not stained with the dye, thus the length of the regrowth measured in centimeters revealed the level of tested cereals tolerance to Al^3+^.

### 2.3. RNA Extraction and Semi-Quantitative Reverse Transcription-PCR (sqRT-PCR)

Total RNA was isolated from liquid N_2_-frozen plant roots using the universal RNA purification kit (EURx, Gdańsk, Poland). To remove contaminating genomic DNA, RNA was treated with RNase-free DNase I (Fermentas/Thermo Scientific, Waltham, MA, USA). The RNA quantity was measured spectrophotometrically (NanoDrop ND-1000; Thermo Scientific). The mRNA levels of the two triticale PhyCys (*TrcC-8*, *TrcC-9*) were analyzed after 12, 24, and 48 h from the application of Al^3+^ using the Titanium One-Step RT-PCR Kit (Clontech Laboratories, Mountain View, CA, USA). The oligonucleotide primer sequences used in the sqRT-PCR were as follows: *TrcC-8F*: 5′-CTCTAGCCCTCCTCTTCCTC-3′, *TrcC-8R*: 5′-GGCTGCTAGATTCGTCATGC-3′, *TrcC-9F*: 5′-AGAACGACCTCGAGACCATC-3′, *TrcC-9R*: 5′-GCTTGAATTCCTGGAGCTG-3′, *18S rRNA-F*: 5′-GATCCATTGGAGGGCAAGTC-3′, *18S rRNA-R*: 5′-GATGGCTTGCTTTGAGCACTC-3′. To ensure that equal amounts of the RNA templates were added to each reaction, amplification of the reference *18S rRNA* gene was performed. sqRT-PCR conditions for the amplification of particular phytocystatins, and *18S rRNA* gene were as follows: 60 min at 50 °C and 5 min at 94 °C; 26 cycles (*TrcC-8*) or 25 cycles (*TrcC-9*) or 9 cycles (*18S rRNA*) of 30 s at 94 °C, 30 s at 58 °C (*TrcC-8*) or 60 °C (*TrcC-9*) or 64 °C (*18S rRNA*), 45 s at 68 °C and a final 2 min at 68 °C. Three repetitions of sqRT-PCR amplification, based on the cDNA that was obtained from three independent RNA extractions, were performed. Amplified products were electrophoresed on a 1.2% (*w*/*v*) agarose gel in 1 × TBE running buffer (89 mM Tris, 89 mM boric acid, 2 mM EDTA; pH 8.3) and visualized by SimplySafe (EURx) (Supplementary Figure S1). The densitometric analysis of the bands was performed using the program ImageJ 1.53m version (U. S. National Institutes of Health, Bethesda, MD, USA).

### 2.4. Root Extract Preparation for Determination of Protein Content and Azocaseinolytic and Gelatinolytic Activities

Extracts were obtained by the grinding of 100 mg root samples in a mortar with liquid N_2_ and 1 mL an ice-cold 50 mM 3-(*N*-morpholino)propanesulfonic acid extraction medium (pH 7.2) containing 5 mM β-mercaptoethanol, 2% (*w*/*v*) polyvidone, 5 mM calcium chloride and 0.5% Triton X-100 surfactant. Homogenates were centrifuged (4 °C, 20 min, 16,000× *g*) and extracts were collected.

### 2.5. Protein Assay

Protein concentration was measured in extracts using Bradford reagent (Sigma–Aldrich) and bovine serum albumin as a standard.

### 2.6. Azocaseinolytic Activity Assay

The azocaseinolytic method for the determination of overall protease activity was used [36]. Measurements were performed at pH 5.2 viz. under optimal conditions for cysteine endopeptidases. The reaction mixtures (50 μL extract, 300 μL 100 mM acetic buffer (pH 5.2), 150 μL 0.7% (*w*/*v*) azocasein with 5 mM β-mercaptoethanol) were placed at 37 °C for 2 h. Reactions were terminated by 12% (*w*/*v*) C_2_HCl_3_O_2_. Next, 20 min incubation in an ice-bath was performed and centrifugation of samples were done (4 °C, 15 min, 16,000× *g*). The absorbance of clear supernatants was measured at λ = 340 nm. The one arbitrary unit of azocaseinolytic activity was termed as 0.01 increase of noted absorbance after 1 h of enzymatic reaction per gram of fresh shoot weight (FW) [U h^−1^ g^−1^ FW].

### 2.7. Zymography of Gelatinolytic Activity

Zymography for protease activity was performed according to Labudda et al. [6]. Proteins (60 μg) were electrophoresed with sodium dodecyl sulphate−polyacrylamide gel electrophoresis (Mini-Protean Electrophoresis System, Bio-Rad Hercules, CA, USA). 4% (*w*/*v*) stacking and 12% (*w*/*v*) running gel copolymerized with gelatin (0.1%, *w*/*v*) was used. Renaturation of proteins after electrophoresis was performed by washing the gels in 2.5% (*v*/*v*) Triton X-100 for 30 min. The incubation of gels (18 h, 37 °C) carried out in 0.1 M acetic buffer (pH 5.2) with 5 mM β-mercaptoethanol. Next, gels were put in 0.1% (*w*/*v*) Amido Black in 7% (*v*/*v*) CH_3_COOH and destained with 7% (*v*/*v*) CH_3_COOH. The Spectra™ Multicolor Broad Range Protein Ladder (Thermo Scientific) was used to estimate protein molecular weight. The densitometric analysis of the bands was performed using the program ImageJ 1.53m version (U.S. National Institute of Health).

To verify the participation of cysteine endopeptidases in the response of plant roots to Al^3+^, gels were preincubated with inhibitor for cysteine endopeptidases (EC 3.4.22) 10 μM L-trans-epoxysuccinyl-leucylamido(4-guanidino)butane (E-64) for 30 min on ice-bath. Then, 5 mM β-mercaptoethanol was added and other procedures were the same as for zymography without inhibitor.

### 2.8. Root Structural Studies

#### 2.8.1. Light Microscopy

Root tips (each collected in ten independent randomly selected repeats *n* = 10 from different plants) were fixed in a mixture of 4% (*w*/*v*) paraformaldehyde (Sigma–Aldrich) and 5% (*v*/*v*) glutaraldehyde (Sigma–Aldrich) in 0.1 M sodium cacodylate buffer (pH 7.2) (Sigma–Aldrich) and 1% (*v*/*v*) dimethyl sulfoxide (Sigma–Aldrich) for 10 h at room temperature. Next, samples were rinsed four times in 0.1 M sodium cacodylate buffer (pH 7.2) and post-fixed in osmium tetroxide (0.05%) for 2 h at 4 °C. After 3 times washing in 0.1 M sodium cacodylate buffer (pH 7.2), the specimens were dehydrated in in a graded ethanol series (10, 30, 50 and 70% (*v*/*v*)) for two times by 30 min at 4 °C. For better venting, samples were stored for several days at 4 °C in 70% (*v*/*v*) ethanol solution and finally further dehydrated in 90%, 96% and 99.8% ethanol series, substituted by propylene oxide and infiltrated and embedded in grade medium of epoxy resin (Sigma–Aldrich; equivalent to Epon 812) according to the manufacturer’s instructions. The specimens were polymerized at 65 °C for 16 h. Samples were sectioned on a Leica RM2165 microtome (Leica Microsystems, Wetzlar, Germany) and sections (3 µm thick) were collected on glass slides (Menzel-Gläser, Braunschweig, Germany). For general histology, the semi-thin sections were stained with an aqueous solution of crystal violet dye (1%, Sigma–Aldrich) and examined under an Olympus AX70 “Provis” light microscope (Olympus, Tokyo, Japan) equipped with an Olympus DP50 digital camera (Olympus).

#### 2.8.2. Transmission Electron Microscopy (TEM)

Ultrathin sections (90 nm thick) were taken with a Leica UCT ultramicrotome (Leica Microsystems) and stained with saturated solution of methanolic solution of 0.5% (*w*/*v*) uranyl acetate (Sigma–Aldrich) followed by incubation in 0.5% (*w*/*v*) lead citrate (Sigma–Aldrich). Examinations were made on an FEI 268D “Morgagni” transmission electron microscope (FEI Company, Hillsboro, OR, USA) equipped with an Olympus-SIS “Morada” digital camera (Olympus). Samples were collected in five independent randomly selected repeats.

### 2.9. Statistical Analysis

Representative data collected from at least three biological replicates were shown as means ± confidence intervals. Results were subjected to one-way analysis of variance. The significant differences were estimated using Tukey’s honestly significant difference procedure at *p* < 0.05. Statistical analysis was performed with a free 30-day trial 19.2.02 version of Statgraphics 19 (Statgraphics Technologies, Inc., The Plains, VA, USA).

## 3. Results

### 3.1. Al^3+^ Tolerance Test

**Figure 1:**
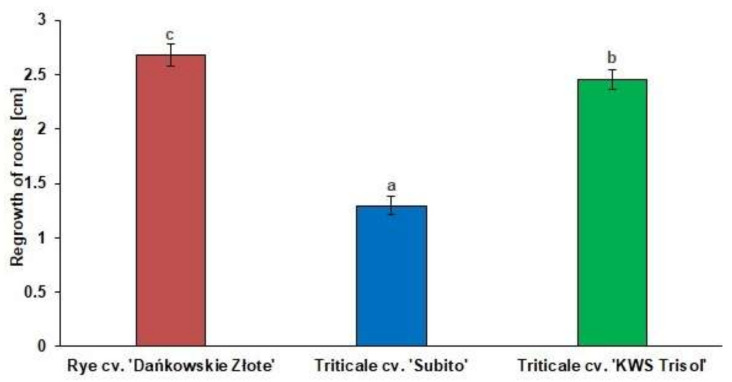
The regrowth of cereal roots after treatment with Al^3+^ (0.59 mM) for 24 h. Results are shown as means ± confidence intervals. Different letters indicate significant differences among values after one-way analysis of variance and Tukey’s honestly significant difference procedure at *p* < 0.05.

The physiological test of Al^3+^ tolerance showed significant differences in the length of the regrowth of roots between rye and triticale cultivars (Figure 1). Average length of root regrowth of rye cv. “Dańkowskie Złote”, triticale cv. “Subito” and triticale cv. “KWS Trisol” were as follows: 2.6 cm, 1.4 cm, and 2.5 cm, respectively (Figure 1), thus rye and “KWS Trisol” triticale have been considered to be tolerant genotypes and “Subito” triticale as a sensitive genotype to Al^3+^ toxicity.

### 3.2. Gene Expression of PhyCys (TrcC-8 and TrcC-9) in the Response of Cereal Roots to Al^3+^

**Figure 2:**
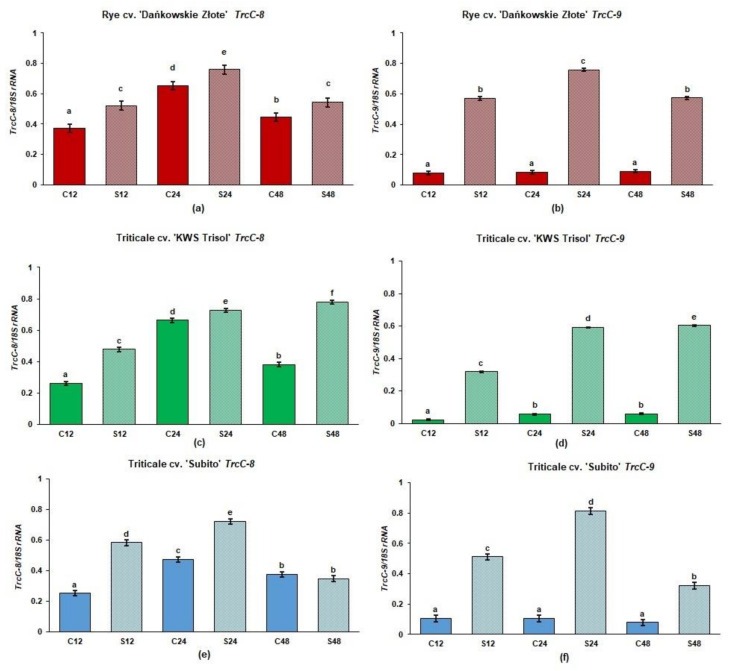
The gene expression of phytocystatins in roots of tolerant rye cv. “Dańkowskie Złote”, tolerant triticale cv. “KWS Trisol” and sensitive triticale cv. “Subito” at 12, 24 and 48 h after treatment with Al^3+^ (0.59 mM) (S) and in untreated plants (C). (**a**) “Dańkowskie Złote” *TrcC-8*; (**b**) “Dańkowskie Złote” *TrcC-9*; (**c**) “KWS Trisol” *TrcC-8*; (**d**) “KWS Trisol” *TrcC-9*; (**e**) “Subito” *TrcC-8*; (**f**) “Subito” *TrcC-9*. Figure shows relative expression (ratios of *TrcC-8* and *9* to *18S rRNA*) of phytocystatin genes after densitometric analysis of the bands. Results are shown as means ± confidence intervals. Different letters indicate significant differences among values after one-way analysis of variance and Tukey’s honestly significant difference procedure at *p* < 0.05.

The level of *TrcC-8* mRNA was higher in rye and triticale roots under Al^3+^ stress than in the untreated controls (Figure 2a,c,e). In the Al^3+^ treated roots of tolerant rye and tolerant triticale “KWS Trisol” the highest level of *TrcC-8* mRNA was observed at 24 and 48 h after Al^3+^ application respectively, while in the treated roots of sensitive triticale “Subito” after 24 h. The level of *TrcC-8* mRNA was similar in the treated and control roots of “Subito” after 48 h (Figure 2e). In turn, the levels of *TrcC-9* mRNA were incredibly low in all untreated controls (Figure 2b,d,f). In rye, the transcript level of *TrcC-9* significantly increased under stress at all sampling points, and it was the highest at 24 h. In tolerant triticale, the expression of *TrcC-9* was enhanced at 12, 24 and 48 h in treated roots and it was the highest at 48 h. In the roots of sensitive triticale, the expression of *TrcC-9* was also enhanced at 12, 24 and 48 h in treated roots and it was the highest at 24 h in treated roots (Figure 2f).

### 3.3. Spectrophotometric and Zymographic Profiling of Proteolytic Activity in Roots of Rye and Triticales under Al^3+^

**Figure 3:**
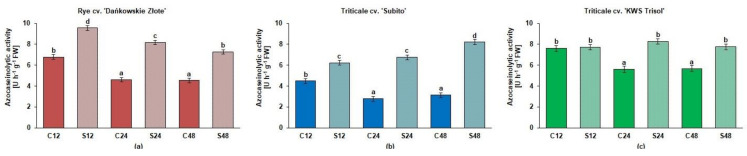
The azocaseinolytic activity at pH 5.2 [U h^−1^ g^−1^ fresh shoot weight FW] in root extracts of cereals at 12, 24 and 48 h after treatment with Al^3+^ (0.59 mM) (S) and in untreated plants (C). Results are shown as means ± confidence intervals. Different letters indicate significant differences among values after one-way analysis of variance and Tukey’s honestly significant difference procedure at *p* < 0.05. (**a**) tolerant rye cv. “Dańkowskie Złote”; (**b**) sensitive triticale cv. “Subito”; (**c**) tolerant triticale cv. “KWS Trisol”.

Overall, in rye and both triticale cultivars, the azocaseinolytic activity at pH 5.2 was higher in roots treated with Al^3+^ than in the untreated controls but with one an exclusive exception when this activity was at a comparable level in treated and control roots of tolerant triticale at 12 h (Figure 3).

**Figure 4:**
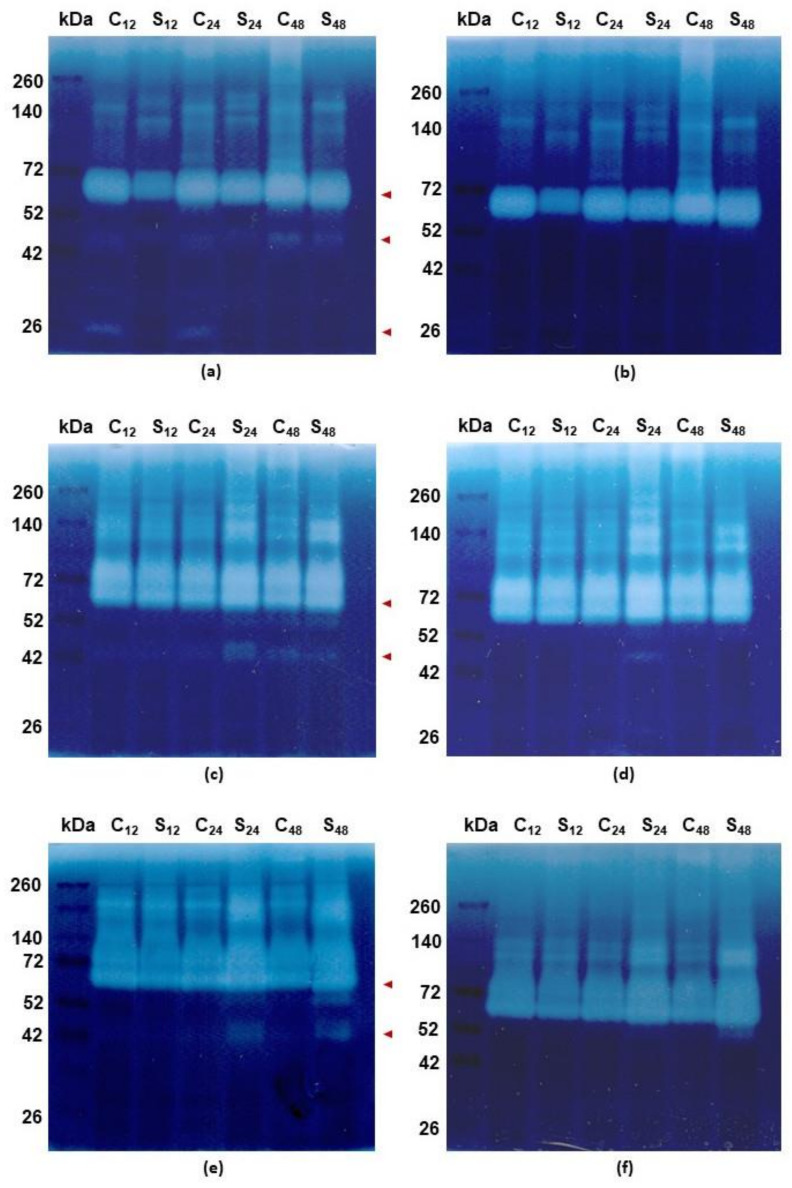
Representative results of the zymographic detection of gelatinolytic activity in roots of cereals at 12, 24 and 48 h after treatment with Al^3+^ (0.59 mM) (S) and in untreated plants (C). Proteins (60 µg) were electrophoresed with sodium dodecyl sulphate–polyacrylamide gel electrophoresis with gelatin as a substrate. Bands of the gelatinolytic activity were visualized by Amido Black. Spectra™ Multicolor Broad Range Protein Ladder was used to determine the protein molecular weight in kilodalton (kDa). (**a**) Electrophoresed tolerant rye cv. “Dańkowskie Złote” protein root samples incubated at pH 5.2 without inhibitor for cysteine endopeptidases 10 μM L-trans-epoxysuccinyl-leucylamido(4-guanidino)butane (E-64); (**b**) Electrophoresed tolerant rye cv. “Dańkowskie Złote” protein root samples incubated at pH 5.2 with E-64; (**c**) Electrophoresed tolerant triticale cv. “KWS Trisol” protein root samples incubated at pH 5.2 without E-64; (**d**) Electrophoresed tolerant triticale cv. “KWS Trisol” protein root samples incubated at pH 5.2 with E-64; (**e**) Electrophoresed sensitive triticale cv. “Subito” protein root samples incubated at pH 5.2 without E-64; (**f**) Electrophoresed sensitive triticale cv. “Subito” protein root samples incubated at pH 5.2 with E-64. The red arrowheads show the bands that pay special attention (for more details including densitometric analysis see text).

The zymographic profiling of gelatinolytic proteolytic activity at pH 5.2 has shown the most proteolytic activities of rye roots between 140 and 42 kDa (Figure 4a). However, lower weight bands at 26 kDa were also observed. The highest gelatinolytic activity was observed at 72 kDa and the activity of these bands was 40%, 10% and 20% lower in samples treated with Al^3+^ (respectively at 12, 24 and 48 h) in comparison to respective untreated controls. Other bands of proteolytic activity were observed between 52 and 42 kDa and they indicated 100%, 38% and 25% more intense activity in untreated controls than in Al^3+^ treated ones (respectively at 12, 24 and 48 h). In the case of bands at 26 kDa the activity was observed only in untreated controls and the activity was 16% higher at 12 h than at 24 h (Figure 4a). The bands of proteolytic activity between 52 and 42 kDa and at 26 kDa were inhibited by specific cysteine endopeptidase inhibitor (10 µM E-64) (Figure 4b).

In the case of tolerant triticale, the bands at 72 kDa were 9% more intense at 12 h in untreated control than in treated roots while at 24 and 48 h the activity level was respectively 15% and 5% higher in the treated roots than in the control (Figure 4c). The bands of additional proteolytic activity between 52 and 42 kDa were clearly observed in untreated control roots at 48 h and in treated roots at 24 and 48 h. The activity of these bands was 35% and 40% lower at 48 h (in untreated and treated roots, respectively) than at 24 h (Figure 4c) and they were inhibited by 10 µM E-64 (Figure 4d).

The gelatinolytic activity bands of sensitive triticale at 72 kDa were 20% more intense at 24 h and 48 h in treated roots than in untreated controls while at 12 h the activity level was 10% lower compared to the control (Figure 4e). The proteolytic activity bands between 52 and 42 kDa were exclusively observed in treated roots at 24 and 48 h. The activity level was 10% lower at 48 than at 24 h. (Figure 4e). These activity bands were inhibited by 10 µM E-64 such as in rye roots (Figure 4f).

### 3.4. Structural Changes Observed under Light Microscope in Roots of Rye and Triticales during Al^3+^ Stress

**Figure 5:**
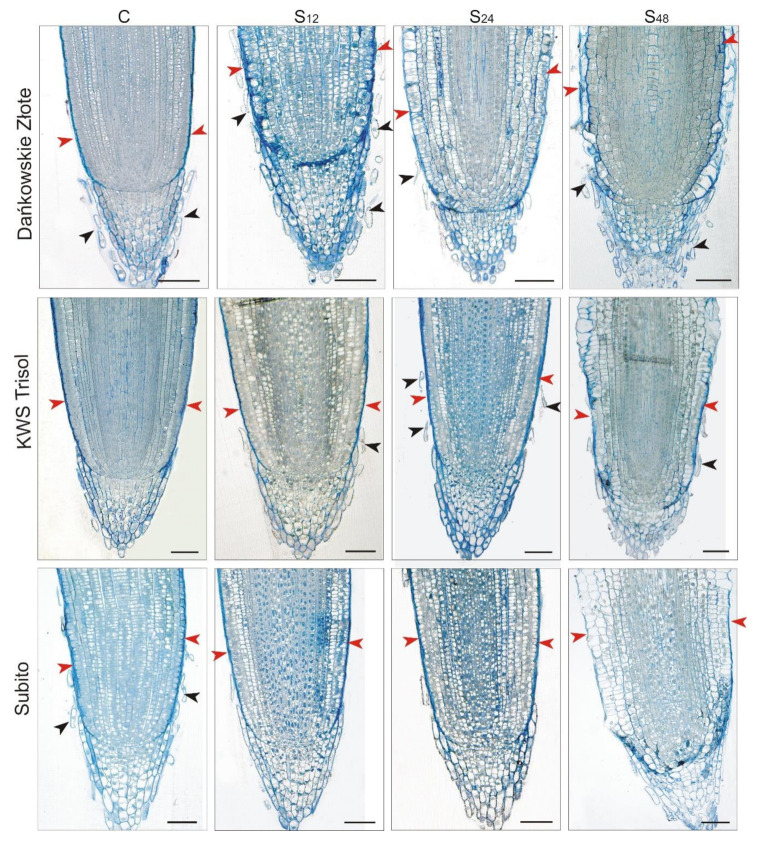
Representative results of the anatomical structure of root tips of tolerant rye cv. “Dańkowskie Złote”, tolerant triticale cv. “KWS Trisol” and sensitive triticale cv. “Subito” at 12, 24 and 48 h after treatment with Al^3+^ (0.59 mM) (S) and in untreated plants (C). Roots sections were stained with an aqueous solution of crystal violet. The anatomical structure of root tips did not change in different sampling points in untreated plants, so only one representative control is shown (C). The red arrowheads show the rhizoderm, while the black ones root border cells. Bar = 100 µm.

Light microscope analysis of the longitudinal section of division zone of rye-treated roots revealed intense degradation of rhizoderm cells at 48 h in comparison to untreated roots (Figure 5). Interestingly, such a change was not visible in the case of the analyzed triticale cultivars. Looking at the anatomical structure of the root tips, particular attention is drawn to the structure of the rye root caps, both in the control and all treated plants. The root caps of the control rye plants were surrounded by mucilage, which could not be observed both in the treated rye roots as well as in the control and treated triticales. Root border cells (RBCs) that are detached from the caps were visible in the mucilage surrounding the caps of the control root rye. In addition, RBCs were also consistently visible in samples showing treated rye roots. In the case of both cultivars of triticale, RBCs are noticeable in both the control and treated plants, but in comparison to rye, their appearance was incidental (Figure 5).

### 3.5. Structural Changes Observed under Transmission Electron Microscope in Roots of Rye and Triticales during Al^3+^ Stress

**Figure 6:**
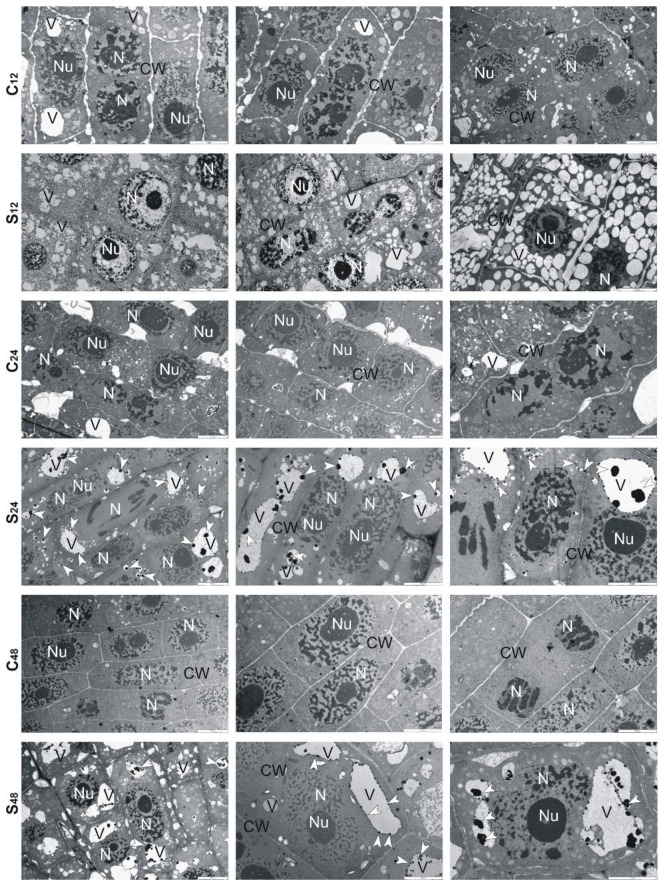
Representative results of the ultrathin sections taken from the roots of tolerant rye cv. “Dańkowskie Złote” at 12, 24 and 48 h after treatment with Al^3+^ (0.59 mM) (S) and in untreated plants (C). Caption: arrowheads: electron-dense precipitate, CW: cell wall, N: nucleus, Nu: nucleolus, V: vacuole. Bar = 5 µm.

**Figure 7:**
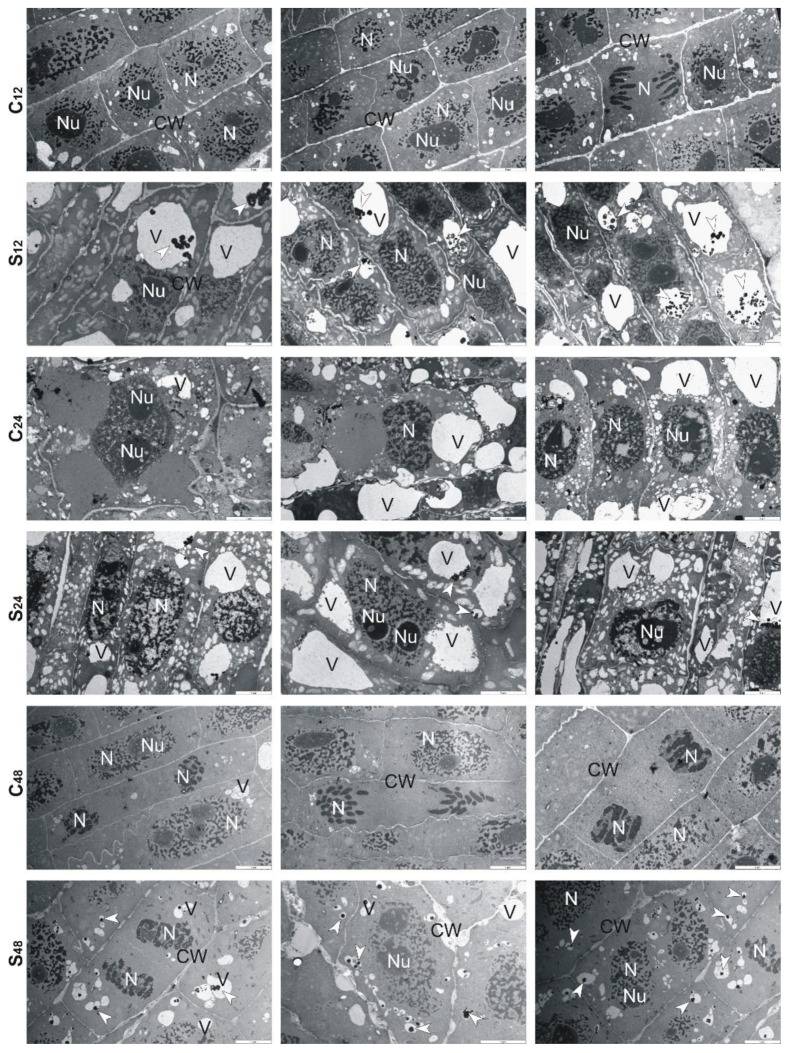
Representative results of the ultrathin sections taken from the roots of tolerant triticale cv. “KWS Trisol” at 12, 24 and 48 h after treatment with Al^3+^ (0.59 mM) (S) and in untreated plants (C). Caption: arrowheads: electron-dense precipitate, CW: cell wall, N: nucleus, Nu: nucleolus, V: vacuole. Bar = 5 µm.

**Figure 8:**
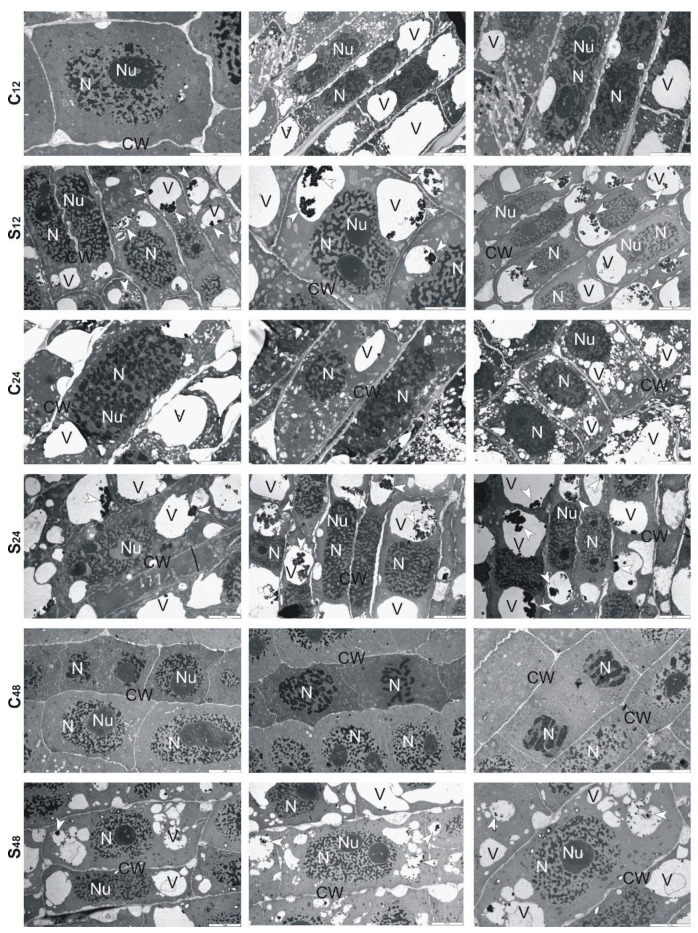
Representative results of the ultrathin sections taken from the roots of sensitive triticale cv. “Subito” at 12, 24 and 48 h after treatment with Al^3+^ (0.59 mM) (S) and in untreated plants (C). Caption: arrowheads: electron-dense precipitate, CW: cell wall, N: nucleus, Nu: nucleolus, V: vacuole. Bar = 5 µm.

Ultrastructural analysis of treated rye roots showed that cells presented thickening of their walls and numerous small vacuoles in comparison to untreated roots (Figure 6). At 24 h, in the center of these vacuoles, electron-dense precipitates were observed, while at 48 h fewer fine vacuoles and precipitates in them were found. Moreover, the cell walls were no longer swollen, and divisions of cells took place (Figure 6). In the treated root cells of tolerant triticale, at 12 and 24 h, numerous small and large vacuoles with electron-dense precipitates were found (Figure 7). Thickened cell walls and fewer minor vacuoles with electron-dense precipitates were visible at 48 h (Figure 7). In sensitive triticale, in treated roots, many large vacuoles with electron-dense precipitates and less swollen cell walls were observed at 12, 24 and 48 h in comparison to untreated ones (Figure 8).

## 4. Discussion

During evolution, plants developed several tolerance mechanisms against Al^3+^. Some of these defense mechanisms are specific to Al^3+^ and some are related to a more general stress response. Our research performed on the molecular, structural, and physiological levels revealed some acclimatizational responses of tolerant rye cultivar, and next they were compared with the responses of two contrasting triticale cultivars.

Applying light microscopic observations of division zones of roots, we noted punctate rhizodermal degradation in Al^3+^ treated rye and a much lower degree in tolerant triticale. In the case of sensitive triticale, no degradation of rhizoderm cells was observed. Delisle et al. [37], within 8 h of exposure to Al, observed a punctate cell death in the root epidermis of tolerant wheat. They have proposed a tolerance mechanism in wheat based on accelerated turnover of rhizodermal cells. Thus, it seems that also in Al^3+^ tolerant rye and tolerant triticale plants, cell death is aimed at replacing epidermal cells intoxicated with Al^3+^ while simultaneous maintaining root growth.

An interesting observation, which seems to indicate one of the factors determining the increased tolerance of rye to Al^3+^ toxicity, concerns structural, and thus also functional (but not investigated in this article) changes in the rye root caps. We observed that the root caps of the control rye were surrounded by mucilage. In addition, RBCs were visible in the mucilage surrounding the caps of the control rye roots. RBCs were also present in samples showing treated rye roots at 12, 24 and 48 h. Cai et al. [38] and Yang et al. [39] have suggested that RBCs and mucilage favor increased Al^3+^ tolerance in rice and pea plants, respectively. According to more recently published data by Nagayama et al. [40] it can be concluded that Al^3+^-binding mucilage produced by RBCs can protect rice root tips from Al^3+^-induced damage. Translating this into our observations, it can be suggested that the presence of mucilage in the area around the RBCs in control rye plants, in a way, constitutively determines rye’s increased tolerance to Al^3+^ stress at the beginning. As RBCs are also seen in root rye samples after treatment, we indicate their involvement in the defense mechanisms of rye against Al^3+^ stress.

To learn more about the structural changes induced by Al^3+^ in cereale root cells, we decided to implement TEM. Cereals often use Al^3+^ exclusion strategy to avoid their over-uptake and limit the transport of Al^3+^ from root to shoot. These plants also try to retain toxic ions in the cell walls, through this reduce the possibility of complexing with cellular macromolecules [41]. The analyzes performed by us showed that at 12 h, the root cells treated with Al^3+^ had swollen cell walls. Perhaps it was because of the accumulation of Al-chelating exudates in the cell walls. Our observations are consistent with results presented by Yang et al. [42]. These authors proved that the cell wall polysaccharides (pectin, hemicellulose 1, and hemicellulose 2) were idiosyncratically involved in the exclusion of Al^3+^ from the root apex of rice plants, and what is important such biochemical response was exclusive to Al^3+^ stress compared with other metals (CdCl_2_, LaCl_3_, and CuCl_2_). Therefore, based on the results of Yang et al. [42] and ours, it can be suggested that the cell walls fulfill an important role in excluding Al^3+^ from roots of cereals.

Moreover, it is worth noting that Sharma et al. [43] indicate vacuole compartmentation as a key player in maintaining HMs homeostasis. We observed changes in the shape and size of the vacuoles. In Al-treated rye at 12 h, there were many small vacuoles, while in the following sampling points, both small and large vacuoles with Al-complexes were visible. More large than small vacuoles were also observed in the root cells of both triticale cultivars. In the root cells of 10-day-old *A. thaliana*, a single enlarged vacuole was observed after treatment with Cd, apparently because of vesicle fusion. Cd-induced formation of small vesicles was enhanced in suspension-cultured cells of *Nicotiana tabacum* [44]. Cd treatment had no effect on vacuoles of radish leaves [45]. No changes were also observed in the case of *Chlamydomonas acidophila* single-cell algae treated with Cu ions. The above studies allow a conclusion that the changes in vacuolar forms depend on the species and type of the metal ion. Both in rye and triticale, in the following hours of the experiment, we observed more large vacuoles than small ones. According to Sharma et al. [43] HM-dependent enhancement in volume of vacuoles successfully leads to the reduction of HMs level in the vacuole lumen and supports trans-tonoplast electrochemical driving force for the sequestration of HMs. The unquestionable benefit of this mechanism is that the HM vacuolar transport can be uninterrupted at a downward energy input with insignificant interference of HMs with other metabolic functions in the vacuoles.

Plants also respond to HMs by triggering the expression of genes that encode proteins involved in stress response [46]. The synthesized proteins are responsible for chelation of metal ions, i.e., phytochelatins, metallothioneins, sequestration of the resulting complexes in vacuoles, and repair or hydrolysis of damaged and no achieved native conformation proteins. We observed that total proteolytic activity increased in Al^3+^-treated roots of the examined plants. It could be related to hydrolyze denatured proteins, which lost their biological function under stress conditions. However, the observed proteolytic activity was the highest in rye at 12 h and decreased in the following sampling points. In turn, in tolerant triticale the level of proteolytic activity was equable in control and stressed conditions while in sensitive one, this activity increased after Al^3+^ treatment. The increase of total proteolytic activity in sensitive triticale roots was also related to the higher activity of cysteine endopeptidases. In Al^3+^ treated roots, at 24 and 48 h, new bands of activity were observed and next reduced by the specific inhibitor for cysteine endopeptidases (E-64). In rye, the bands of cysteine endopeptidase activity were mainly in untreated roots. Although at 48 h, we also observed one activity band in Al^3+^ treated roots. In turn, in treated roots of tolerant triticale the highest activity of cysteine endopeptidases was observed at 24 h and it was lower at 48 h. The activity of cysteine endopeptidases in treated roots may be related to the participation of these enzymes in processes that accompany stress, such as senescence and programmed cell death (PCD) [47,48].

Due to the especially important role of cysteine endopeptidases in protein turnover and involvement in stress-related processes, their uncontrolled activity may threaten the internal proteolytic balance in the cell. Our previous studies have showed that PhyCys effectively control the activity of endogenous cysteine endopeptidases involved in seed development and response to abiotic stress [17,49]. Two of nine identified triticale PhyCys—*TrcC-8* and *TrcC-9*—took part in the response of triticale roots and leaves to drought stress [17]. The present study showed that changes of cysteine endopeptidase activity in Al^3+^-treated roots of rye corresponded to changes of PhyCys gene expression. The expression levels of both genes were the highest at 24 h and decreased at 48 h in treated roots. At the same time, at 48 h, we observed a band of cysteine endopeptidase activity. Perhaps higher activity of cysteine endopeptidases was related to PCD in rhizodermis. In sensitive triticale, PhyCys expression levels decreased while in tolerant ones we observed an increase of these genes’ expression. The observations correspond to lower (10%) and higher (40%) decrease of triticale cysteine endopeptidase activity, respectively. These differences between triticale cultivars indicate a significant role of these inhibitors in Al tolerance. The analysis of the effect of Cd^2+^ and Ni^2+^ on the structure and functioning of the cystatin from *Brassica juncea* indicated an enhancement in inhibitory activity, ∼26% for Ni^2+^ and ∼16% for Cd^2+^. The results suggest alterations in cystatin conformation upon interplay with these HMs [50]. The authors concluded that the increased inhibitory activity of cystatin under HM stress may protect the plant cells from the damage due to the cysteine endopeptidases. However, another feature of this preservation can be the sequestration of HMs by cystatin. The affinity values denoted by Kd (inverse of the binding constant K) for both Cd^2+^-cystatin and Ni^2+^-cystatin interactions indicate this inhibitor as a moderate chelator [50]. Therefore, the regulation of proteolysis positively influences the acclimatization of plants to Al^3+^, and PhyCys constitutes an essential element of this defense strategy.

## 5. Conclusions

The results of our research showed for the first time that accelerated turnover of rhizodermal cells, the presence of root border cells and mucilage in the root tip area (especially close to the caps), vacuolar compartmentalization, and effective regulation of proteolysis are key to maintaining rye growth and development under aluminum stress. Higher tolerance to Al^3+^ of the tolerant triticale cultivar may result from balanced proteolysis and more effective removal of Al^3+^ together with rhizoderm cells. The innovation of our results is that we presented proteolytic changes in rye exposed to Al^3+^ and compared it with the responses of two triticale cultivars differing in Al^3+^ tolerance. We believe that such an approach is utilitarian and perhaps in the future such targeted research may favor modern plant breeding based on molecular biology methods.

However, to better understand the reasons for the observed protein differences between Al^3+^-treated cereals, it is important to conduct further research including Al determination and localization in root compartments and changes in the composition of proteins and their post-translational modifications using proteomic methods.

## Data Availability

All data are available at the corresponding author.

## Supplementary Materials

The following are available online at https://www.mdpi.com/article/10.3390/cells10113046/s1, Figure S1: Representative results of the semi-quantitative reverse transcription-PCR detection of gene expression of phytocystatins and *18S rRNA*.
