# Supplementary material for: Proteolytic and Structural Changes in Rye and Triticale Roots under Aluminum Stress
Source: Cells. 2021 Nov 5;10(11):3046. doi: 10.3390/cells10113046 (PMC8618286; doi:10.3390/cells10113046)
Supplement: Supplementary file 1 [file cells-10-03046-s001.zip › cells-1408623-supplementary.pdf]

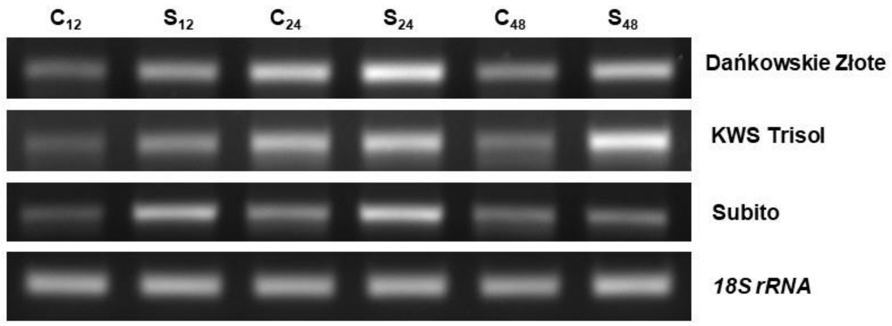

(a)

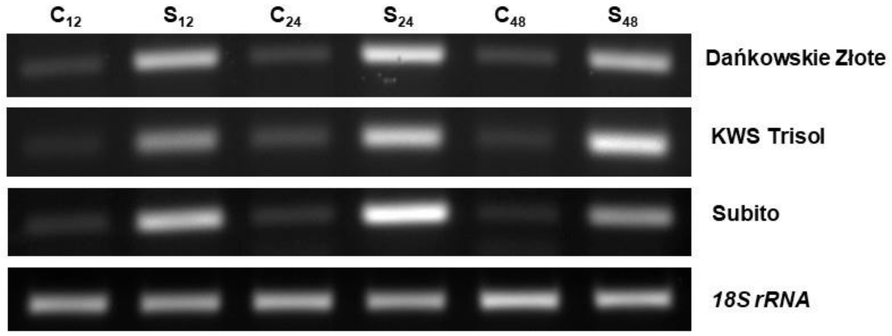

(b)

Figure S1. Representative results of the semi-quantitative reverse transcription-PCR detection of gene expression of phytocystatins and *18S rRNA*. The gene expression patterns of phytocystatins in roots of tolerant rye cv. 'Dańkowskie Złote', tolerant triticale cv. 'KWS Trisol' and sensitive triticale cv. 'Subito' at 12, 24 and 48 h after treatment with Al^{3+} (0.59 mM) (S) and in untreated plants (C). **(a)** *TrcC-8*. **(b)** *TrcC-9*. The amplified products were electrophoresed on an agarose gel and visualized by SimplySafe.
